# Cross-level pathways from teacher autonomy support to adolescent intrinsic motivation through basic psychological need satisfaction: a multilevel mediation model with classroom climate as a boundary condition

**DOI:** 10.3389/fpsyg.2026.1890620

**Published:** 2026-10-26

**Authors:** Weinan Du, Shichang Zhang

**Affiliations:** School of Marxism, Harbin Institute of Technology, Harbin, Heilongjiang, China

**Keywords:** adolescents, basic psychological needs, classroom motivational climate, intrinsic motivation, multilevel structural equation modeling, teacher autonomy support

## Abstract

**Introduction:**

Teacher autonomy support is associated with adolescent intrinsic motivation, but the shared classroom context is often omitted from analyses. Drawing on self-determination theory and achievement goal theory, this study examined indirect associations through autonomy, competence and relatedness need satisfaction and their moderation by classroom motivational climate.

**Methods:**

Cross-sectional questionnaire data were collected from 2,634 students in 72 secondary-school classes across grades 7–12. A two-level structural equation model with random slopes separated within-class and between-class pathways. Measurement invariance was evaluated across three grade bands. Indirect effects and pairwise contrasts were assessed using 20,000 Monte Carlo draws, and mastery and performance climates were tested as cross-level moderators.

**Results:**

Scalar measurement invariance supported pooling the grade bands. All three indirect effects were positive at both levels. Within-class estimates through autonomy, competence and relatedness were 0.148, 0.102 and 0.068, respectively; between-class estimates were 0.256, 0.167 and 0.154. Explained variance in intrinsic motivation was 31% within classes and 47% between classes. Pairwise contrasts favored autonomy over the other needs, with weaker evidence for its advantage over competence; competence and relatedness were not statistically distinguishable. The between-class joint equality test was borderline. Mastery climate strengthened the first-stage autonomy and competence paths, whereas performance climate weakened them. Neither moderator was significant for the relatedness path.

**Discussion:**

The findings support distinguishing individual perceptions from shared classroom conditions when studying autonomy-supportive teaching. Classroom climate may help explain variation in the associations between perceived support, need satisfaction and intrinsic motivation. Because the study was cross-sectional and relied on student self-report, the estimated indirect pathways do not establish causal or temporal ordering.

## 1 Introduction

Adolescence is the developmental window in which intrinsic motivation for academic learning tends to erode rather than consolidate. Longitudinal evidence from secondary-school cohorts documents a steady decline in self-reported interest, curiosity-driven engagement, and willingness to invest sustained effort as students move through the middle and upper grades (Gnambs and Hanfstingl, 2016; Engler and Westphal, 2024). The trend matters because intrinsic motivation, more than externally regulated forms, forecasts deep learning strategies, conceptual transfer, and persistence under academic adversity (Howard et al., 2021). Educators in many East Asian settings confront a particularly sharp version of the problem: high-stakes assessment cultures and densely scheduled classrooms tend to crowd out precisely the conditions under which volitional engagement thrives (Yu et al., 2018a). Published intervention reports suggest, moreover, that the gap between what teachers intend to cultivate and what students actually experience as supportive has widened rather than narrowed (Reeve and Cheon, 2021), and that disjunction is what motivates the present inquiry.

Self-determination theory (SDT) supplies the most coherent lens for examining this gap, and it also explains why we single out one class of teacher behavior. Teachers can nourish students through warmth, through structure, and through autonomy support, and each of the three plausibly contributes to all three basic psychological needs (Jang et al., 2010; Aelterman et al., 2019). Autonomy support nevertheless holds a distinctive position in the theory. It is the behavioral class defined by its effect on the perceived locus of causality, and therefore the class most directly implicated in whether a student experiences schoolwork as self-endorsed or as imposed (Reeve, 2009). Black and Deci (2000) provided the classic demonstration: across a semester of college chemistry, students who perceived their instructor as autonomy-supportive gained in autonomous self-regulation, perceived competence, and interest, and lost anxiety, with the gain in autonomous regulation carrying the effect forward into performance. Autonomy support is also the most malleable of the three behavioral classes under teacher training (Reeve and Cheon, 2021), which gives its study a practical as well as a theoretical warrant.

Pairing autonomy support with intrinsic motivation as the outcome raises a question of conceptual proximity that deserves to be met head-on. The proximity that most needs guarding against is not the one between a teaching behavior and a motivational outcome, which are plainly distinct, but the one between autonomy need satisfaction and intrinsic motivation, since both invoke the vocabulary of self-endorsed action; we therefore treat the satisfied need as an antecedent mechanism rather than a restatement of the outcome, and develop that process rationale in Section 2.1. When self-determined functioning is indexed by the relative autonomy index, interest and enjoyment items enter the composite directly (Ryan and Connell, 1989), so an apparent autonomy advantage can be an artifact of overlapping item content rather than a substantive finding. We therefore keep the two constructs operationally separate, index intrinsic motivation with a dedicated subscale rather than with a relative autonomy composite, report sample items for every scale, and treat the relative ranking of the three mediators as a claim requiring formal statistical contrasts rather than an inspection of point estimates.

A second and more consequential limitation of the existing literature concerns the level at which the process is modeled. Most prior studies treat students as independent observations and estimate the autonomy-support-to-motivation pathway entirely at the individual level, even though students are nested within classes, teachers, and motivational climates (Bardach et al., 2020b). Once variance is partitioned, between-class differences in perceived autonomy support routinely account for a tenth to a fifth of the total, which signals a shared classroom component that single-level models silently fold into within-student perception (Marsh et al., 2012; Lüdtke et al., 2008). Equally overlooked is the moderating role of the classroom motivational climate, the aggregated normative emphasis on mastery as against relative performance, which may sharpen or blunt the translation of individual perceptions into need experiences (Robinson, 2023). Cross-level interactions of this kind have been theorized more often than they have been tested with adequate power and sampling design (Cheon et al., 2023).

The multilevel question carries a cultural one inside it. SDT holds the three needs to be universal nutriments, and meta-analytic evidence from the United States and East Asia supports that claim for autonomy (Yu et al., 2018b; Vite et al., 2024). Chinese studies nevertheless tend to report heavier loadings on relatedness and competence than on autonomy (Zhou et al., 2022), which invites the possibility that universality holds at one level of analysis while cultural inflection appears at another. Because a multilevel design separates the person-specific from the class-shared portion of each construct, it offers a way of putting that possibility to an empirical test rather than leaving it as an interpretive preference.

The present study accordingly fits a two-level structural equation model in which individual perceptions of teacher autonomy support predict intrinsic motivation through the parallel mediation of the three needs, while class-level mastery and performance climates moderate the strength of the mediated paths. Three questions guide the work. Does the tripartite mediation survive once between-class variance is properly partitioned? Which need carries the heaviest mediating weight within classes, and does the ordering shift between them? And does a mastery-oriented climate amplify the link from autonomy support to need satisfaction, producing cross-level moderated mediation? Sampling six consecutive grades serves these questions directly: the analytic unit is the class, so the design needs enough classes to support cross-level interaction tests, and a span that covers the whole of secondary schooling allows us to ask whether the modeled effects hold across the developmental window in which motivation is known to decline. We test that consistency explicitly rather than assume it, through measurement invariance analyses and through grade-level comparisons reported in Section 4. The contribution is threefold: methodologically, we move from single-level to multilevel mediation with explicit boundary testing; theoretically, we sharpen the contextual specification of SDT by treating classroom climate as a substantive moderator rather than a nuisance covariate; and practically, we offer a more granular map of where, and under which climatic conditions, autonomy-supportive practice yields its strongest motivational dividends.

## 2 Theoretical foundation and hypotheses

### 2.1 Self-determination theory and basic psychological need satisfaction

Self-determination theory departs from drive-reduction and operant accounts by treating the person as inherently active, growth-oriented, and predisposed toward integrating new experience into a coherent self (Ryan and Deci, 2017). Its central proposition is that human flourishing depends less on the quantity of motivation than on its qualitative character, which the theory arranges along an autonomy-control continuum running from amotivation through external, introjected, identified, and integrated regulation, and terminating in intrinsic motivation as the prototype of self-determined action (Deci and Ryan, 2000). Intrinsic motivation occupies a special place in that taxonomy because it arises from interest and inherent satisfaction in the activity itself rather than from any separable consequence (Ryan and Deci, 2000, 2020). The theory thereby reframes the educational question: instead of asking how to increase motivation, it becomes more productive to ask how to cultivate its more autonomous, self-endorsed forms.

The engine that converts contextual inputs into motivational quality is the satisfaction of three basic psychological needs. Autonomy concerns the experience of volition and self-endorsement of one's actions; competence refers to the felt capacity for effective interaction with the environment; relatedness captures a sense of mutual care and meaningful connection with others (Chen et al., 2015). Deci and Ryan posit these needs as universal nutriments, necessary for psychological growth across cultures, life stages, and domains, even where their behavioral expressions differ (Vansteenkiste et al., 2020). A subsequent refinement, the dual-process model, distinguishes need satisfaction from need frustration and assigns them asymmetric consequences, with satisfaction fuelling growth and frustration uniquely predicting ill-being and defensive functioning (Vansteenkiste and Ryan, 2013; Rouse et al., 2020). The present study measures satisfaction only, so we note the distinction as a caution against treating need processes as interchangeable and return to it among the limitations.

Locating intrinsic motivation as an outcome of need satisfaction, rather than as a synonym for it, requires an explicit theoretical warrant, and cognitive evaluation theory—the sub-theory of self-determination theory that first specified this process—supplies one (Deci and Ryan, 1985; Ryan and Deci, 2000). The distinction turns on a difference in kind rather than in degree. Need satisfaction is an experiential state: it registers whether a person, while acting, feels volitional, effective, and connected. Intrinsic motivation is a regulatory quality: it describes why the person engages, namely for the inherent interest and enjoyment of the activity itself. That the two are conceptually adjacent, and that autonomy in particular carries shared vocabulary across the pair, does not render them redundant, for a student may feel genuine choice and competence in a task she nonetheless pursues for instrumental reasons, and the reverse can occur as well. The functional claim of cognitive evaluation theory is that events satisfying the needs for autonomy and competence enhance and sustain intrinsic motivation, whereas events that thwart them erode it, with relatedness furnishing the relational security within which that catalysis is more reliably maintained. Seen this way, the three needs are not rival motivational variables competing with the outcome but the proximal psychological nutriments through which a distal contextual input—here, autonomy-supportive teaching—is converted into self-determined engagement. The autonomy that the model treats as a mediator is accordingly the satisfied need, the felt volition of the acting person, and not the autonomous regulation that defines intrinsic motivation at the terminus of the continuum; to conflate the two would be to collapse a mechanism into its own effect. It is precisely this mediator-to-outcome ordering, grounded in the functional logic of cognitive evaluation theory and corroborated by experimental and intervention work in which need-supportive conditions raise intrinsic motivation (Reeve and Cheon, 2021; Cheon et al., 2023), that licenses the process model developed below.

Educational applications of SDT have grown into one of the empirically denser corners of motivational psychology. Classroom studies spanning elementary, secondary, and tertiary settings find that perceived autonomy support from teachers consistently predicts higher autonomy and competence satisfaction, with somewhat looser but still significant links to relatedness (Vansteenkiste et al., 2012; Wang and Hu, 2022; Zhang et al., 2024). The relatedness path appears to run more through a teacher's emotional availability than through the granting of choice as such (Ruzek et al., 2016). Meta-analyses conducted across cultures report that the satisfaction of all three needs is associated with deeper learning strategies, stronger persistence, and more adaptive emotional engagement (Howard et al., 2021; Vasconcellos et al., 2020; Bureau et al., 2022), and syntheses that compare Western with East Asian samples find the autonomy wellbeing association to be of comparable magnitude in both, notwithstanding recurrent claims that autonomy is a culturally parochial concern (Yu et al., 2018b; Vite et al., 2024). Two implications follow for the present study. The three needs warrant simultaneous rather than aggregated modeling, because their differential weights speak to which classroom mechanisms matter most. And because need experiences are intrinsically person-by-context, shaped by what the teacher does and by how the surrounding climate amplifies or muffles those signals, they invite rather than merely permit a multilevel formulation.

### 2.2 The multilevel structure of teacher autonomy support and classroom motivational climate

Teacher autonomy support is best understood not as a global disposition but as a bundle of recognizable instructional acts. Reeve (2009) behavioral specification identifies four: providing meaningful choice within curricular constraints, offering rationales that connect tasks to students' values, acknowledging rather than dismissing negative affect when difficult content is encountered, and refraining from controlling language, deadlines, and pressuring incentives. Each component carries its own causal weight. Choice provision loads most heavily on autonomy satisfaction, rationale giving on identified regulation, affect acknowledgment on relatedness, and the avoidance of control on the prevention of need frustration (Patall et al., 2018; Ahmadi et al., 2023). Treated as a composite, then, autonomy support is theoretically richer than single-item operationalisations suggest, and that granularity matters when one asks what teachers are actually doing in a given room.

A second feature, less often theorized, concerns the level at which autonomy support resides. Although it is measured through individual student reports, perceived autonomy support carries both an idiosyncratic component and a shared, classroom-bound one, because students in the same room are exposed to the same teacher behaviors and to one another's interpretations of them (Wang, 2024). Marsh's framing of contextual variables as possessing distinct within- and between-cluster meanings applies here with particular force (Marsh et al., 2009). A student's report can be split into the class mean, which indexes what the room shares, and the deviation from it, which indexes how that student reads the teacher relative to her classmates. The two components need not behave alike, and treating their sum as a single individual-difference variable risks the aggregation bias that contextual-effects research has documented for three decades (Lüdtke et al., 2008; Marsh et al., 2012).

Beyond teacher behavior itself, the classroom carries a more diffuse contextual signal in its motivational climate. Achievement goal theory distinguishes two prototypical forms: mastery-oriented climates, in which effort, learning, and improvement are normatively rewarded, and performance-oriented climates, in which relative ability and social comparison dominate the evaluative texture of the room (Midgley et al., 2000; Bardach et al., 2020a). The mechanism that connects climate to need satisfaction is worth stating concretely, because it is what makes climate a moderator rather than merely another predictor. In a mastery-oriented room, errors are treated as information about where to go next, feedback is referenced to a student's own prior work, and tasks are assigned for what they teach rather than for how they sort. Under those norms, a teacher's offer of choice reads as genuine devolution of control and a rationale reads as an invitation to endorse the task, so the same behavior delivers more autonomy satisfaction. In a performance-oriented room, where evaluation is public and comparative, the identical offer of choice is plausibly read as another occasion on which one's standing will be judged, and the informational content of the teacher's behavior competes with an evaluative signal that undercuts felt volition (Jiang and Zhang, 2021; Zheng et al., 2023). Climate, on this account, calibrates how teacher behavior is decoded before it ever reaches the needs.

The nested structure of classrooms also shapes the arithmetic of the individual-context interaction. Because students are non-randomly assigned to teachers, share peer norms, and accumulate co-experienced feedback, their observations are dependent in ways that violate the independence assumption underlying single-level mediation (Warburton, 2017; Ju et al., 2025). Properly modeled, this dependency becomes informative rather than a nuisance: it permits estimation of cross-level interactions in which the slope from autonomy support to need satisfaction varies as a function of class-aggregated climate. Effects of this kind, modest as they often are in raw magnitude, can substantially reorganize where and for whom autonomy-supportive teaching produces its largest motivational dividends (Wang et al., 2022).

### 2.3 Multilevel mediation pathways and hypothesis development

The decomposition sketched above forces the autonomy-support-to-motivation chain to be specified at two levels, and the substantive hypotheses follow from that requirement. Within classes, a student who perceives more autonomy support than her classmates should report greater satisfaction of the three needs and, in turn, more interest-driven engagement with academic tasks, because the person-specific deviation captures a dyadic attunement between that student and her teacher. Between classes, a room whose teacher is consistently autonomy-supportive should show a higher class mean on each need and a higher class mean on motivation, because consistency across occasions is what allows a teaching style to register as a shared classroom property rather than as an idiosyncratic experience. The two paths are conceptually distinct, and contextual-effects theory gives no reason to expect them to be equal in size (Marsh et al., 2012; Dong and Wu, 2025). Specifying this chain as a mediation commits us to a directional reading—autonomy support acts on motivation by first changing what students experience of their needs—that follows from the functional logic set out in Section 2.1 and from experimental self-determination research; the cross-sectional design, however, estimates these paths from covariation and cannot by itself fix their temporal order, a caveat we retain among the limitations.

Regarding the relative weight of the three mediators, SDT positions autonomy as the need most proximally addressed by autonomy-supportive behavior, which yields a directional expectation. We state that expectation as a hypothesis while acknowledging its vulnerability: because our outcome lies at the self-determined end of the regulation continuum, the autonomy path enjoys a degree of conceptual proximity that competence and relatedness do not share, so any observed ordering must be defended with formal contrasts and interpreted with that proximity in view. Hypothesis 1 therefore holds that autonomy support exerts a positive indirect effect on intrinsic motivation through all three needs at both levels, with autonomy expected to carry the heaviest within-class weight.

Whether the mediated paths bend under the prevailing climate is a separate question, and the decoding mechanism described in Section 2.2 supplies its rationale. Because a mastery climate strengthens the informational reading of teacher behavior, it should sharpen the conversion of that behavior into need experience; because a performance climate introduces comparative threat, it should dampen the same conversion. Hypothesis 2 accordingly predicts that mastery climate amplifies the slope from autonomy support to need satisfaction, and Hypothesis 3 predicts that performance climate attenuates it. We expect this moderation to be most pronounced for the autonomy need, since the evaluative texture of a room bears most directly on whether an offer of choice is experienced as volition-granting, but we test all three needs so that the expectation is examined rather than assumed. Combined with the mediation in Hypothesis 1, these predictions describe a moderated multilevel mediation in which the magnitude of the indirect effect varies systematically across classrooms. Figure 1 presents the resulting conceptual model, in which rectangles denote manifest variables, ellipses denote latent variables, and the two class-level climate constructs act on the first-stage slope rather than on the mediator directly.

**Figure 1:**
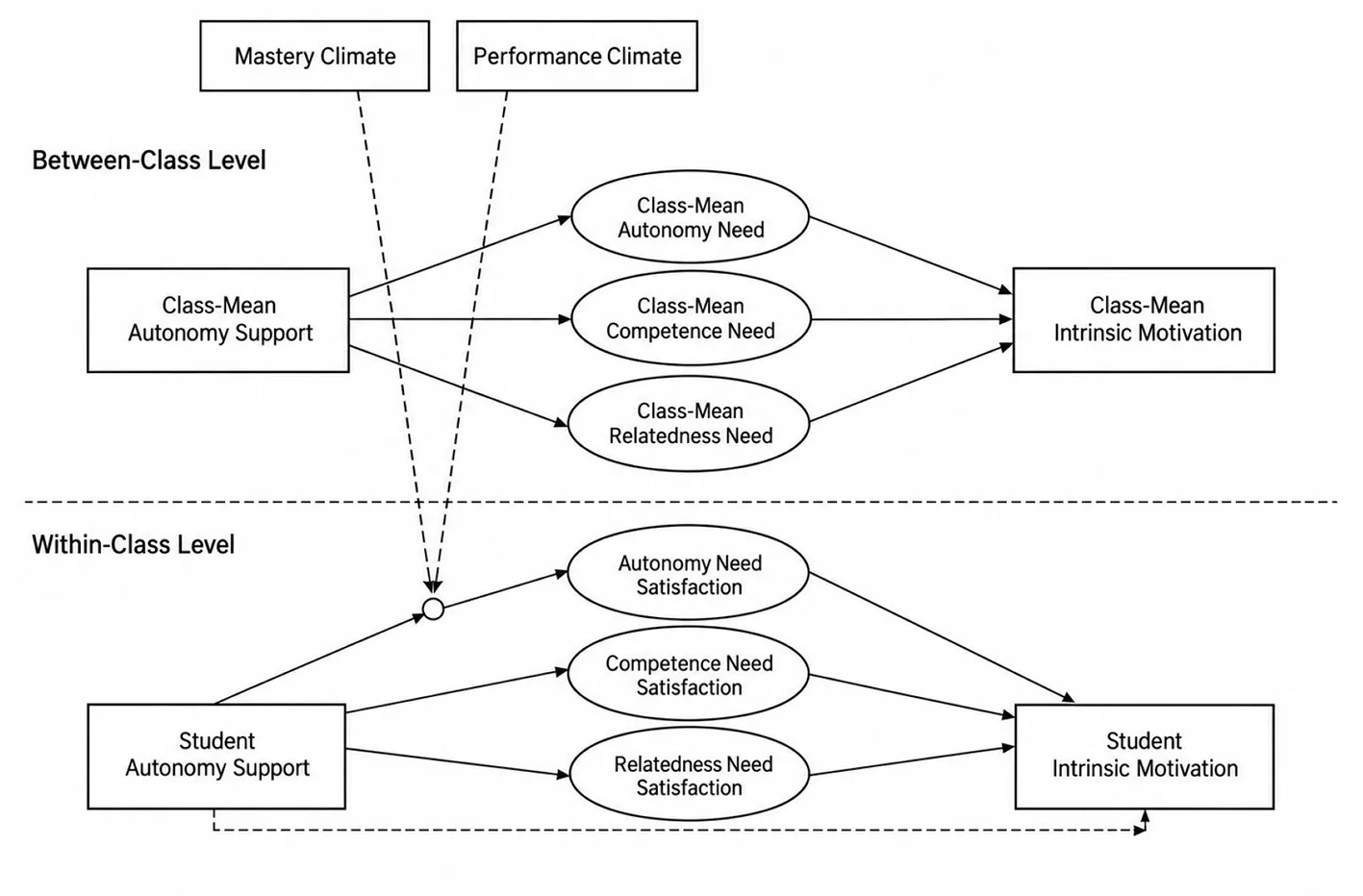
Conceptual model of the moderated multilevel mediation. Teacher autonomy support predicts intrinsic motivation through autonomy, competence, and relatedness need satisfaction at the within-class level and at the between-class level, and the class-aggregated mastery and performance climates moderate the first-stage slope. Rectangles represent manifest variables and ellipses represent latent variables. The diagrams show moderation of the autonomy pathway for clarity; corresponding first-stage moderation models were also fitted for competence and relatedness.

## 3 Research design and methods

### 3.1 Participants and data collection procedure

Participants were recruited through cluster sampling, the appropriate strategy when analytic interest lies in classroom-level variance and intact groups must be preserved (Pituch and Stevens, 2016). Six full-time secondary schools in two prefecture-level cities of north-eastern China agreed to participate, spanning urban core, suburban, and county-affiliated catchment areas so as to widen socioeconomic representativeness. Within each school we drew two intact classes per grade at random from the official class roster, covering grades 7 through 12, which local schools designate as Junior 1 to Senior 3. The final cluster pool comprised *N* = 72 classes, and all enrolled students present on the testing day were invited to participate. Sampling the full secondary span was a deliberate design choice rather than a convenience: the cross-level hypotheses require a sufficient number of Level-2 units, and covering the grades over which motivation is known to decline allows the consistency of the modeled effects across that span to be tested rather than presumed.

After excluding 87 protocols with more than 10% missing items or with invariant response patterns flagged by Mahalanobis distance screening, the analytic sample retained *N* = 2,634 students nested in *N* = 72 classes, with a mean cluster size of *M* = 36.58 (*SD* = 4.30, range 28 to 47). The retained sample was 51.4% female, with a mean age of *M* = 14.82 years (*SD* = 1.76, range 12 to 18). Han ethnicity accounted for 89.6% of respondents, in line with the demographic profile of the sampled region. Father's education was distributed as follows: middle school or below 27.3%, high school or vocational 41.5%, junior college 18.2%, and bachelor's degree or higher 13.0%; the maternal distribution was comparable. Table 1 reports the full demographic breakdown.

**Table 1:** Demographic characteristics of the analytic sample (*N* = 2,634 students nested in *N* = 72 classes).

Variable	Category	Frequency (*n*)	Percentage (%)	Cumulative (%)
Grade level	Junior 1 (G7)	462	17.5	17.5
	Junior 2 (G8)	451	17.1	34.6
	Junior 3 (G9)	438	16.6	51.2
	Senior 1 (G10)	444	16.9	68.1
	Senior 2 (G11)	423	16.1	84.2
	Senior 3 (G12)	416	15.8	100.0
Sex	Female	1,354	51.4	51.4
	Male	1,280	48.6	100.0
School type	Urban key	906	34.4	34.4
	Urban ordinary	981	37.2	71.6
	Suburban or county-affiliated	747	28.4	100.0

Percentages are rounded to one decimal place and may not sum to exactly 100 because of rounding.

Grade-level coverage is balanced across the six cohorts, which keeps the multilevel estimates from being confounded with developmental skew. Surveys were administered during a regular class period of 35 min, with one trained graduate research assistant per classroom reading the standard instructions aloud and remaining available for clarification. Homeroom teachers were absent during administration, a precaution recommended in school-based survey work to mitigate evaluation apprehension and reduce socially desirable responding about teacher behavior (Podsakoff et al., 2003). Booklets were distributed in pre-randomized order, sealed in opaque envelopes on completion, and transported to a central data center for double-entry verification.

The protocol was reviewed and approved by the Institutional Review Board of the first author's home institution (approval number HIT-MARX-IRB-2024-EDU-037), consistent with national guidelines for educational research with minors (Wang et al., 2024). Because participants were minors, written informed consent was obtained in advance from a parent or legal guardian, and student assent was confirmed verbally on the day of administration. Students were told that they could withdraw at any point without academic consequence and that responses would be linked only by anonymised class-student codes (Pan and Yao, 2023). No clinical procedures or health-related endpoints were involved; the inquiry was wholly educational in character and was therefore conducted under the institutional review framework rather than clinical-trial reporting standards.

### 3.2 Measurement instruments and variable operationalization

Four self-report instruments were administered, each adapted from established sources and re-validated with the present sample. Teacher autonomy support was assessed with a revised Learning Climate Questionnaire (LCQ-R), retaining 8 of the original 15 items judged most pertinent to the four behavioral facets set out in Section 2.2, namely choice, rationale, affect acknowledgment, and non-controlling language (Williams and Deci, 1996; Simon and Salanga, 2021; Monacis et al., 2023). Because a rationale-giving item alone would represent the construct too narrowly, we report two sample items spanning the facets: “My homeroom teacher lets me decide how to go about my schoolwork whenever that is possible” for choice provision, and “My homeroom teacher offers me reasons for the rules and expectations in class” for rationale giving. Of the eight retained items, three tap choice and student decision-making, two tap rationale giving, two tap acknowledgment of students' perspectives and feelings, and one taps the absence of controlling language. Responses were given on a 7-point Likert scale anchored at 1 = strongly disagree and 7 = strongly agree.

Basic psychological need satisfaction was measured with the satisfaction subscales of the Basic Psychological Need Satisfaction and Frustration Scale, using 4 items per need and 12 items in total (Chen et al., 2015). Sample items read “I feel a sense of choice and freedom in the things I undertake at school” (autonomy), “I feel confident that I can do things well in my schoolwork” (competence), and “I feel that the people I care about at school also care about me” (relatedness). Academic intrinsic motivation was assessed with the intrinsic-motivation subscale of the Academic Motivation Scale, High School version, comprising 4 items whose stem was reframed for general schoolwork rather than a specific course (Vallerand et al., 1992; Stover et al., 2012), an instrument whose intrinsic-motivation subscale has accumulated the strongest validity evidence among the AMS dimensions (Fairchild et al., 2005); a sample item reads “Because I experience pleasure and satisfaction while learning new things.” Classroom motivational climate was captured by the mastery-oriented (6 items) and performance-oriented (5 items) subscales of the Patterns of Adaptive Learning Scales, written in plural-perception form so as to elicit a class-level referent (Midgley et al., 2000); sample items read “In our class, really understanding the material is what matters most” and “In our class, it matters a great deal who gets the highest scores.” All instruments were forward- and back-translated by two bilingual researchers, with discrepancies resolved through expert consensus in line with standard cross-cultural adaptation procedure (Algharaibeh, 2021).

Reporting the item content matters for more than transparency. Section 2.1 has already established, on theoretical grounds, why autonomy need satisfaction and intrinsic motivation occupy different positions in the self-determination process—the former a psychological nutriment, the latter a regulatory quality—so the concern here is the complementary, empirical one of whether their item content keeps them separable in this data set. Because autonomy satisfaction and intrinsic motivation sit close together on the self-determination continuum, and because the relative autonomy index folds interest items into the autonomy composite (Ryan and Connell, 1989), readers need to be able to judge whether the two constructs are empirically separable in this data set. The item wording above keeps them distinct in content, with the autonomy items referring to volition and self-endorsement and the motivation items referring to interest and enjoyment in learning, and the discriminant-validity evidence reported next supports that separation.

Confirmatory factor analyses estimated separately for each instrument supported the intended structures. All McDonald's ω coefficients exceeded 0.80, all composite reliabilities exceeded 0.80, and all average variance extracted values exceeded 0.50, evidencing acceptable internal consistency and convergent validity against conventional benchmarks (Tavakol and Wetzel, 2020). Discriminant validity was confirmed by the Fornell-Larcker criterion, the square root of every average variance extracted exceeding the absolute correlations with adjacent constructs. The pairing of most concern is the one just described: the square root of the average variance extracted was 0.77 for autonomy need satisfaction and 0.81 for intrinsic motivation, both above their mutual correlation of *r* = 0.51, and the heterotrait-monotrait ratio for that pair was 0.62, comfortably below the 0.85 benchmark. The two constructs are therefore treated as distinct in what follows.

Three class-level variables, autonomy support, mastery climate and performance climate, were specified to operate at the class level through aggregation of student responses to the class mean, which is the operationalisation implied by the plural-referent item stem. Whether such aggregation is psychometrically defensible was tested through three indices (Scalas et al., 2014). The intraclass correlation *ICC*(1) quantifies the proportion of total variance located between classes and is defined, in the random-intercept null model, as the ratio of the between-class variance component to the sum of the between- and within-class components. The reliability of the class mean as an estimator of its latent class-level construct is indexed by *ICC*(2), and interrater consensus relative to a uniform null distribution is gauged by the within-group agreement statistic *r*_*wg*(*j*)_. Defensible aggregation conventionally requires *ICC*(1)≥0.05, *ICC*(2)≥0.70, and a median *r*_*wg*(*j*)_≥0.70, although the second threshold is somewhat lenient when class sizes are moderate (LeBreton and Senter, 2008).

Table 2 shows that all three class-level constructs cleared the conventional thresholds, with *ICC*(1) between 0.13 and 0.21, values at the upper end of what classroom research typically reports, and *ICC*(2) comfortably above 0.80 given the average cluster size of roughly 37 (Shao et al., 2025). The median *r*_*wg*(*j*)_ was at least 0.78 for every class-level scale, so aggregation to the Level-2 referent was retained for all three constructs. It is worth registering how consequential these values are for the wider literature. Contextual-effects work has shown that ignoring a shared component of this size produces biased estimates of group-level effects and understated standard errors (Lüdtke et al., 2008; Marsh et al., 2012), and the values reported here sit well above the threshold at which that bias becomes material. Yet single-level analyses of autonomy support remain common even where the design is manifestly nested: studies of teacher support in intact secondary classes have modeled student reports as independent observations without partitioning class variance (Xu et al., 2023; Pan and Yao, 2023), and others have reported classroom-referenced constructs without documenting either the intraclass correlation or the agreement statistics that would justify aggregation (Zhou et al., 2022). The present design was built to avoid that omission.

**Table 2:** Aggregation diagnostics for class-level variables.

Class-level variable	ICC(1)	ICC(2)	Median rwg(j)	Decision
Teacher autonomy support (LCQ-R)	0.18	0.89	0.84	Aggregate retained
Mastery climate (PALS)	0.21	0.91	0.81	Aggregate retained
Performance climate (PALS)	0.13	0.85	0.78	Aggregate retained
Autonomy need satisfaction (referent comparison)	0.07	0.74	0.71	Individual-level retained
Competence need satisfaction (referent comparison)	0.06	0.71	0.69	Individual-level retained

LCQ-R, revised Learning Climate Questionnaire; PALS, Patterns of Adaptive Learning Scales. Values are reported to two decimal places.

One further precaution follows from the age range of the sample. Because the analytic sample spans early to late adolescence, the modeled associations can be compared across grades only if the instruments carry the same meaning across them. We therefore tested measurement invariance for all four instruments across three grade bands, grades 7 to 8, grades 9 to 10, and grades 11 to 12, proceeding from configural through metric to scalar models and judging each step by the change in comparative fit index and root mean square error of approximation rather than by the chi-square difference alone, given its sensitivity to sample size (Chen, 2007). Results appear in Section 4.1. Common method bias was screened with Harman's single-factor test, which extracted no dominant factor, the largest eigenvalue accounting for 28.4% of total variance.

### 3.3 Multilevel structural equation modeling and analytic strategy

The analytic engine is a two-level structural equation model estimated in Mplus 8.10 with full-information maximum likelihood and robust standard errors, an estimator that accommodates the mild non-normality typical of Likert data and absorbs missingness under the assumption that data are missing at random (Muthén and Muthén, 2017). Multilevel structural equation modeling was preferred over conventional multilevel regression because it separates measurement error from structural variance and permits simultaneous estimation of within- and between-class paths, a property essential to the contextual-effect logic developed in Section 2.2 (Preacher et al., 2010; Marsh et al., 2009). Table 3 collects the symbols that recur through this section and the next, so that the equations below can be read without repeated glossing; equations are presented once and referred to descriptively thereafter rather than by number.

**Table 3:** Notation key for the multilevel mediation model.

Symbol	Level	Meaning
$AS_{ij}^{w}$	1	Group-mean-centered student perception of autonomy support
*AS*_·*j*_	2	Class-aggregated autonomy support
*N*_*kij*_	1	Need satisfaction, with k denoting autonomy, competence, or relatedness
*IM*_*ij*_	1	Student-reported intrinsic motivation
*Mas*_*j*_	2	Class-aggregated mastery climate
*Per*_*j*_	2	Class-aggregated performance climate
$a_{kj}^{w}$	random slope	Within-level path from autonomy support to need k
$b_{k}^{w}$	1	Within-level path from need k to intrinsic motivation
$u_{kj}^{o}$, $u_{j}^{IM}$, *u*_*akj*_	2	Class-level random intercepts and slope residuals
ρ_*IM*_	model level	Indirect effect of interest

The measurement model begins from the observation that a student's report of autonomy support decomposes additively into the class mean and the individual deviation from it, which is the standard within-between separation adopted in multilevel structural equation modeling. Writing that decomposition explicitly gives


$$\begin{array}{c} AS_{ij}=AS_{\cdot j}+\left(AS_{ij}-AS_{\cdot j}\right)=AS_{ij}^{B}+AS_{ij}^{W} \end{array}$$

where the first term captures the class-shared component obtained by aggregation and the second the person-specific deviation. Group-mean centring of all Level-1 predictors and grand-mean centring of all Level-2 predictors followed the recommendations of Enders and Tofighi (2007) for keeping cross-level interactions interpretable.

Building on that decomposition, the within-level mediator and outcome equations take the form


$$\begin{array}{l} N_{kij}=\alpha_{kj}+a_{kj}^{W}\;AS_{ij}^{W}+r_{kij}^{N}\\ IM_{ij}=\beta_{0j}+\underset{k}{\sum }b_{k}^{W}\;N_{kij}+{c\prime }^{W}\;AS_{ij}^{W}+r_{ij}^{IM} \end{array}$$

in which the summation runs over the three needs and the residual direct path is denoted by the primed coefficient. This is a 1-1-1 mediation: predictor, mediators, and outcome are all measured and modeled as Level-1 constructs after group-mean centring, so the within-level indirect effect captures how individual-difference variation in perceived autonomy support, net of the shared classroom signal, propagates through need satisfaction to motivation (Preacher et al., 2010).

The class-shared component requires a different specification, because the predictor is now a Level-2 construct while the mediators and outcome remain at Level-1, which makes the design a 2-1-1 mediation operating through the Level-2 random intercepts. Those intercepts are modeled as


$$\begin{array}{l} \alpha_{kj}=\gamma_{k0}+a_{k}^{B}\;AS_{\cdot j}+u_{kj}^{0}\\ \beta_{0j}=\delta_{00}+\underset{k}{\sum }b_{k}^{B}\;\alpha_{kj}+{c\prime }^{B}\;AS_{\cdot j}+u_{j}^{IM} \end{array}$$

with the class-level disturbances collected in the final terms of each expression. Two implications attach. The within- and between-level structural paths need not be equal, and indeed contextual-effect logic predicts that they often differ; and a class-mean effect on motivation may persist even where the corresponding within-level path is small (Marsh et al., 2012).

To let the strength of the first stage vary across rooms, the within-level slope is treated as random and modeled as an outcome of the two climate variables, which is the slopes-as-outcomes specification


$$\begin{array}{c} a_{kj}^{W}=\gamma_{ak0}+\gamma_{ak1}\;Mas_{j}+\gamma_{ak2}\;Per_{j}+u_{akj} \end{array}$$

so that the conversion of perceived autonomy support into need satisfaction bends with the class climate. Substituting this conditional slope into the within-level indirect effect yields a climate-conditioned mediated effect equal to the product of the conditional first-stage slope and the second-stage path, whereas the between-level indirect effect collapses to the simple product of the corresponding Level-2 paths. Together these expressions operationalise Hypotheses 1 through 3.

Because such products are non-normally distributed in finite samples, inference relies on Monte Carlo confidence intervals rather than the Sobel approximation. Following Preacher and Selig (2012), we drew 20,000 random samples of the constituent path estimates from a multivariate normal distribution with means and covariance matrix supplied by the model output, and took the empirical 2.5th and 97.5th percentiles of the resulting product distribution as the 95% interval; an indirect effect is treated as supported when that interval excludes zero (Tofighi and MacKinnon, 2011).

Comparing mediators demanded an additional step that the original analysis plan did not contain. Ranking point estimates cannot establish that one mediator outperforms another, so we formed the pairwise differences between the three indirect effects within the same 20,000 Monte Carlo draws, preserving the covariance among the constituent paths, and treated a difference as significant only when its interval excluded zero (Preacher and Hayes, 2008). A simultaneous Wald test of the joint hypothesis that the three indirect effects are equal was computed from the same draws, and an equivalent equality-constrained model was estimated as a cross-check. The same logic was applied separately at the within- and between-class levels.

Model fit was judged against the conventional thresholds of a comparative fit index at or above 0.90, a root mean square error of approximation at or below 0.08, and within- and between-level standardized root mean square residuals at or below 0.08, recognizing that the between-level residual is the more sensitive index of class-level misfit (Hu and Bentler, 1999). Measurement invariance across grade bands was evaluated with the criteria of Chen (2007), a change in comparative fit index no greater than 0.010 and a change in root mean square error of approximation no greater than 0.015 between nested models. Sensitivity analyses included re-estimation under Bayesian Markov chain Monte Carlo with diffuse priors as a cross-check on the maximum-likelihood solution (Muthén and Asparouhov, 2012), together with a specification in which the between-level paths were constrained to equal their within-level counterparts, rejection of that constraint being taken as evidence of contextual asymmetry between levels. Figure 2 traces the full analytic sequence, from the class-level predictor and climate moderators down to the individual-level mediator-outcome chain, using the same notation as Figure 1.

**Figure 2:**
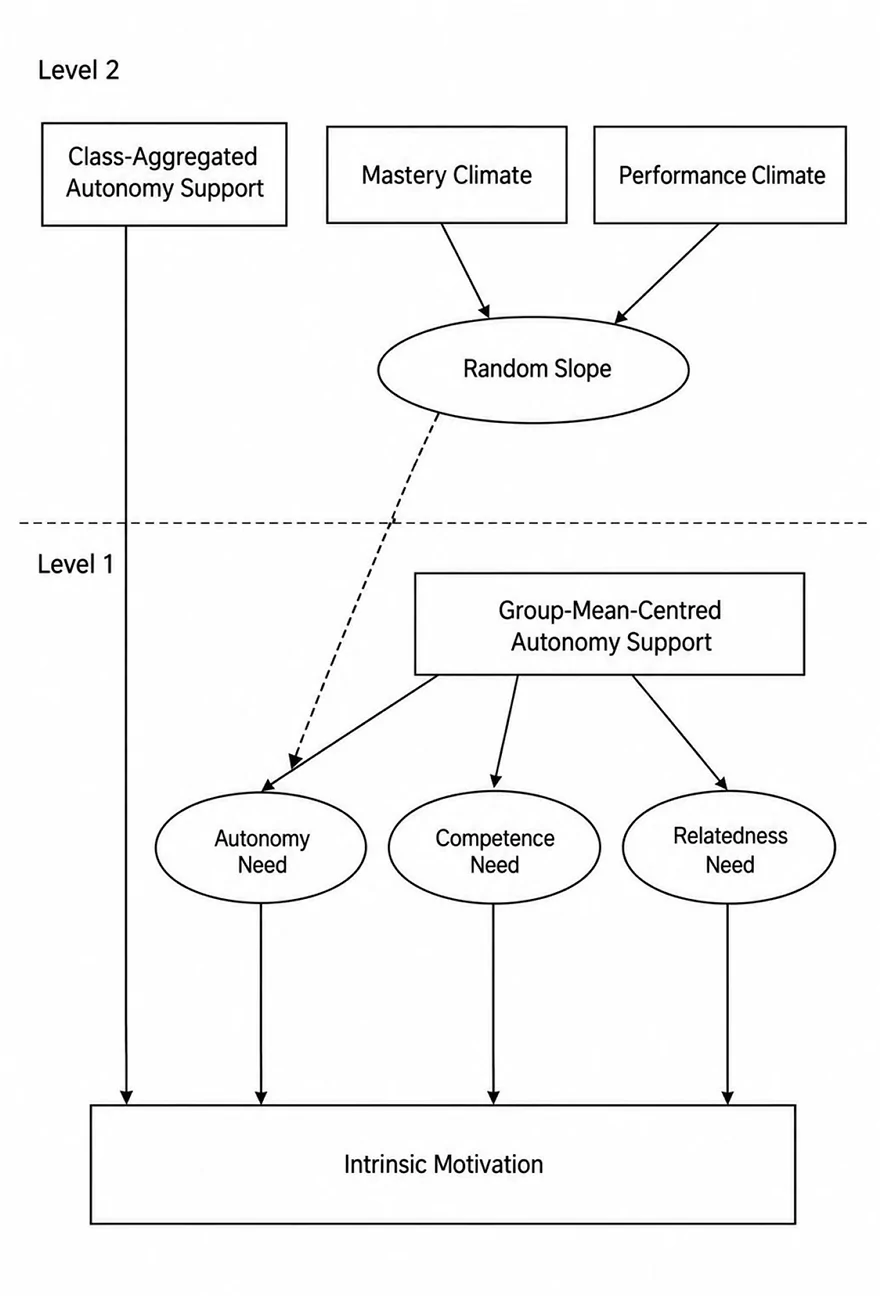
Path diagram of the estimated moderated multilevel mediation model. The within-level slope of teacher autonomy support on each need satisfaction is allowed to vary across classes and is modeled as a function of the class-aggregated mastery and performance climates. The figure depicts the analytic specification rather than the conceptual model, and follows the notation of Figure 1, with rectangles for manifest variables and ellipses for latent variables. The diagrams show moderation of the autonomy pathway for clarity; corresponding first-stage moderation models were also fitted for competence and relatedness.

## 4 Results

### 4.1 Descriptive statistics, measurement invariance, and common method bias diagnostics

Across the seven focal variables, sample means clustered toward the upper-middle range of their respective scales, with autonomy support (*M* = 4.82, *SD* = 1.13 on a 1 to 7 metric) and competence need satisfaction (*M* = 3.62, *SD* = 0.78 on a 1 to 5 metric) showing the most pronounced positive shifts. Skewness ranged from −0.61 to 0.34 and kurtosis from −0.52 to 0.91, comfortably within the boundaries that justify robust maximum-likelihood estimation rather than a more elaborate non-normality correction (Kline, 2016). Bivariate correlations behaved in the directions predicted by SDT: autonomy support correlated positively with each of the three need satisfactions, with *r* between 0.38 and 0.46, and with intrinsic motivation, *r* = 0.42, while performance climate was inversely associated with autonomy need satisfaction, *r* = −0.21. Table 4 summarizes these values. All coefficients in that table are zero-order correlations computed at the individual level on the full sample of *N* = 2,634 students, without decomposition into within- and between-class components; the level-specific associations are reported in Section 4.2.

**Table 4:** Means, standard deviations, skewness, and individual-level zero-order correlations among study variables (*N* = 2,634).

Variable	*M*	*SD*	Skew	1	2	3	4	5	6	7
1. Autonomy support	4.82	1.13	−0.41	-						
2. Autonomy need satisfaction	3.45	0.81	−0.18	0.46	-					
3. Competence need satisfaction	3.62	0.78	−0.27	0.39	0.52	-				
4. Relatedness need satisfaction	3.71	0.74	−0.61	0.38	0.48	0.45	-			
5. Intrinsic motivation	4.58	1.06	−0.33	0.42	0.51	0.49	0.44	-		
6. Mastery climate	3.58	0.69	−0.22	0.34	0.31	0.29	0.27	0.30	-	
7. Performance climate	3.04	0.72	0.12	−0.18	−0.21	−0.15	−0.12	−0.14	−0.09	-

Correlations were computed at the individual level (N = 2,634 students). Autonomy support and intrinsic motivation were rated on a 1 to 7 scale and all other variables on a 1 to 5 scale. Every reported correlation is significant at *p* < 0.01.

Because the sample spans six grades, the invariance of the four instruments across that span was examined before any structural model was fitted. Configural models fitted acceptably in all three grade bands, and the imposition of equal loadings and then equal intercepts produced changes in fit that stayed inside the criteria of Chen (2007) for every instrument, as Table 5 reports. Scalar invariance therefore held, which licenses both the pooling of the six cohorts in a single model and the comparison of latent means across grades reported below.

**Table 5:** Measurement invariance of the four instruments across three grade bands (grades 7 to 8, 9 to 10, and 11 to 12).

Instrument	*ΔCFI* (metric)	*ΔRMSEA* (metric)	*ΔCFI* (scalar)	*ΔRMSEA* (scalar)	Decision
LCQ-R (autonomy support)	−0.003	0.002	−0.005	0.004	Scalar invariance supported
BPNSFS satisfaction subscales	−0.004	0.003	−0.006	0.005	Scalar invariance supported
AMS intrinsic motivation	−0.002	0.001	−0.004	0.003	Scalar invariance supported
PALS mastery and performance	−0.005	0.004	−0.008	0.006	Scalar invariance supported

Values are changes relative to the preceding, less restricted model: the metric columns compare metric with configural models and the scalar columns compare scalar with metric models. Criteria follow Chen (2007): the absolute value of Δ*CFI* must not exceed 0.010 and that of Δ*RMSEA* must not exceed 0.015. BPNSFS, Basic Psychological Need Satisfaction and Frustration Scale; AMS, Academic Motivation Scale.

Mean perceived autonomy support declined across grades, with seventh-graders reporting the highest values (*M* = 5.04, *SD* = 1.08) and twelfth-graders the lowest (*M* = 4.62, *SD* = 1.16). A one-way analysis of variance with grade as the factor confirmed that the differences were statistically significant, *F*(5, 2628) = 9.84, *p* < 0.001, η^2^ = 0.018, and Tukey comparisons located the contrast principally between the junior and senior bands. The trend was not strictly linear: mean autonomy support rose slightly from Grade 9 to Grade 10 before declining again. Figure 3 displays the grade-by-grade pattern.

**Figure 3:**
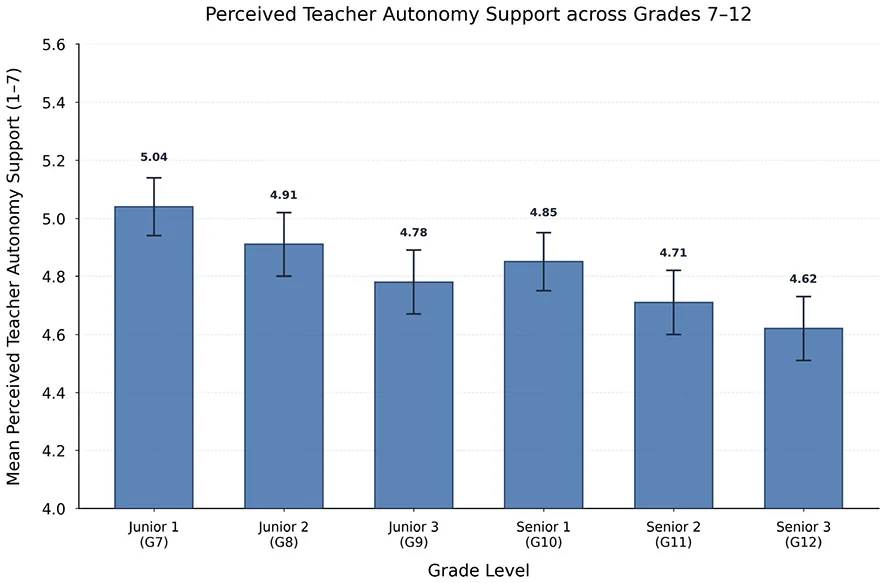
Mean perceived teacher autonomy support across grades 7 to 12, with error bars representing 95% confidence intervals. The decline is interrupted at the junior-to-senior transition, indicating a non-linear developmental trajectory.

A six-factor confirmatory model, comprising autonomy support, the three needs, intrinsic motivation, and a method-effects factor for negatively worded items, fitted acceptably, with χ^2^/*df* = 2.84, CFI = 0.953, TLI = 0.946, RMSEA = 0.043 (90% CI [0.041, 0.045]), and SRMR = 0.038; all standardized loadings fell between 0.62 and 0.86 (Tavakol and Wetzel, 2020). Composite reliabilities ranged from 0.83 to 0.92. Common method bias was then assessed in two ways. Harman's single-factor solution explained 28.4% of total variance, well below the 40% threshold usually taken as cause for concern (Podsakoff et al., 2003). The latent-method-factor approach added an unmeasured method factor onto which all indicators loaded; the resulting improvement in fit was modest, ΔCFI = 0.012 and ΔRMSEA = 0.004, and method-factor loadings averaged 0.18 (Liang et al., 2007). We therefore proceeded to the multilevel analyses without correction.

### 4.2 Multilevel mediation tests and cross-level path analysis

Before structural estimation, an unconditional null model partitioned outcome variance across the two levels. The intraclass correlations were 0.14 for intrinsic motivation, 0.13 for autonomy need satisfaction, 0.10 for competence, and 0.12 for relatedness. All four exceed the intraclass-correlation threshold of 0.05, so an individual-level analysis alone would have biased standard errors and understated the contextual share of variance (Snijders and Bosker, 2012). Roughly one-seventh of the variance in motivation lies between classrooms rather than within them.

The 1-1-1 within-class mediation paths returned a coherent triplet. Group-mean-centered autonomy support predicted each need positively: autonomy, $a_{A}^{W}=0.41$ (*SE* = 0.03, *p* < 0.001); competence, $a_{C}^{W}=0.34$ (*SE* = 0.03, *p* < 0.001); and relatedness, $a_{R}^{W}=0.31$ (*SE* = 0.03, *p* < 0.001). Each need in turn predicted intrinsic motivation, with $b_{A}^{W}=0.36$, $b_{C}^{W}=0.30$, and $b_{R}^{W}=0.22$, all *p* < 0.001. Monte Carlo simulation with 20,000 draws yielded within-level indirect effects of 0.148, 95% CI [0.116, 0.181], through autonomy; 0.102, 95% CI [0.077, 0.130], through competence; and 0.068, 95% CI [0.047, 0.092], through relatedness. None of the three intervals straddled zero, so all three within-level mediated paths were retained (Bauer et al., 2006).

The 2-1-1 between-class mediation, which treats class-aggregated autonomy support as the predictor and the random intercepts of the needs as Level-2 mediators, returned uniformly larger coefficients. Class-mean autonomy support predicted class-mean autonomy ($a_{A}^{B}=0.61$), competence ($a_{C}^{B}=0.49$), and relatedness ($a_{R}^{B}=0.55$), each *p* < 0.001, and the corresponding paths to class-mean intrinsic motivation were $b_{A}^{B}=0.42$, *b*^*B*^ = 0.34, and $b_{R}^{B}=0.28$. The resulting between-level indirect effects were 0.256, 95% CI [0.192, 0.327], through autonomy; 0.167, 95% CI [0.116, 0.223], through competence; and 0.154, 95% CI [0.100, 0.213], through relatedness. A constraint test in which the within- and between-level structural paths were forced to equality produced a substantial decrement in fit, Δχ^2^(6) = 31.42, *p* < 0.001, which supports the contextual asymmetry anticipated within the framework of Marsh et al. (2006). Figure 4 displays the two sets of indirect effects side by side.

**Figure 4:**
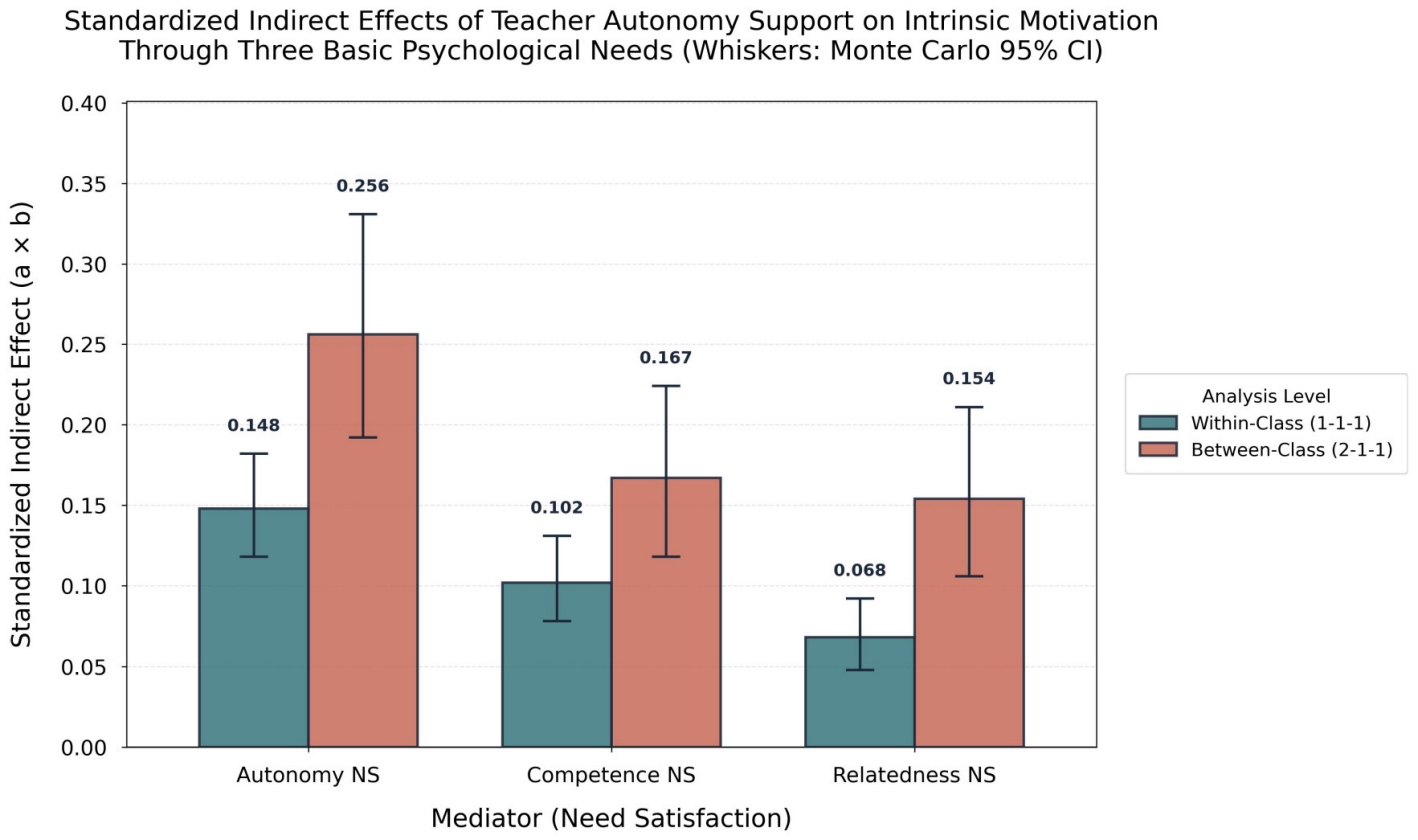
Standardized indirect effects of teacher autonomy support on intrinsic motivation through autonomy, competence, and relatedness need satisfaction, displayed separately at the within-class (1-1-1) and between-class (2-1-1) levels. Whiskers represent Monte Carlo 95% confidence intervals.

Whether the ordering visible in Figure 4 constitutes a difference in kind rather than in point estimate was then tested directly. Table 6 reports the three pairwise contrasts at each level, computed from the same 20,000 Monte Carlo draws so that the covariance among constituent paths is preserved. Within classes, the autonomy path exceeded the relatedness path by a clear margin and exceeded the competence path only marginally, while competence and relatedness could not be distinguished. Between classes, the picture was weaker still: autonomy again exceeded both of the other mediators, but the margins were narrow, and the difference between competence and relatedness was negligible. A simultaneous test of the equality of the three indirect effects was significant within classes, χ^2^(2) = 15.96, *p* < 0.001, but only borderline between them, χ^2^(2) = 5.89, *p* = 0.053, and the equality-constrained model reproduced the same conclusion.

**Table 6:** Monte Carlo contrasts between the three indirect effects at the within- and between-class levels (20,000 draws).

Level	Contrast	Difference	95% Monte Carlo CI	*p*
Within class	Autonomy vs. competence	0.046	[0.004, 0.088]	0.031
	Autonomy vs. relatedness	0.079	[0.040, 0.118]	< 0.001
	Competence vs. relatedness	0.034	[−0.001, 0.069]	0.053
Between class	Autonomy vs. competence	0.089	[0.003, 0.178]	0.043
	Autonomy vs. relatedness	0.102	[0.015, 0.192]	0.022
	Competence vs. relatedness	0.013	[−0.068, 0.091]	0.761

Differences are expressed in the metric of the standardized indirect effects. Intervals excluding zero indicate a statistically significant difference between the two mediators.

Three competing specifications were then estimated for comparative fit. Model A retained only the direct effect of autonomy support on intrinsic motivation at both levels and produced AIC = 51,428, BIC = 51,486, and RMSEA = 0.072. Model B inserted a single composite need-satisfaction mediator and improved to AIC = 50,914, BIC = 50,990, and RMSEA = 0.058. Model C, the full parallel multiple-mediator specification with three separate needs at both levels, yielded the best indices, with AIC = 50,612, BIC = 50,728, CFI = 0.948, RMSEA = 0.046, SRMR-within = 0.039, and SRMR-between = 0.061 (MacKinnon et al., 2007). The likelihood-ratio test favored Model C over Model B, Δχ^2^(8) = 287.40, *p* < 0.001. Explained variance in motivation was *R*^2^ = 0.31 at the within level and *R*^2^ = 0.47 at the between level.

### 4.3 Cross-level moderation by classroom motivational climate and boundary conditions

The moderation tests built on the random-slope specification set out in Section 3.3. Two slopes-as-outcomes models were estimated: the first allowed the slopes from autonomy support to each need satisfaction to vary as a function of the two climate moderators, and the second extended the same logic to the second-stage paths from need satisfaction to intrinsic motivation. For the first stage, the estimated slope variance was τ_*a*_ = 0.05, χ^2^(71) = 198.70, *p* < 0.001, confirming meaningful between-class heterogeneity in how strongly student-perceived autonomy support converts into need satisfaction (Bauer et al., 2006).

Because all three needs mediate the focal relation, the moderation analysis was run for all three rather than for autonomy alone. Table 7 reports the results. Mastery climate amplified the first-stage slope for autonomy and, more weakly, for competence, and performance climate attenuated both; neither moderator reached significance for relatedness. This gradient matches the expectation set out in Section 2.3, while leaving open why relatedness proved insensitive to climate in these data.

**Table 7:** Cross-level moderation of the first-stage slope from teacher autonomy support to each need satisfaction.

Mediator	Mastery climate (γ_*a*1_)	*p*	Performance climate (γ_*a*2_)	*p*	Index of moderated mediation (mastery)
Autonomy need satisfaction	0.13	< 0.001	−0.09	0.004	0.061 [0.034, 0.094]
Competence need satisfaction	0.09	0.006	−0.06	0.041	0.027 [0.008, 0.049]
Relatedness need satisfaction	0.06	0.087	−0.04	0.190	0.013 [−0.004, 0.031]

Coefficients are unstandardised slopes-as-outcomes estimates. Indices of moderated mediation are reported with Monte Carlo 95% confidence intervals in brackets.

For the autonomy need, which carried the strongest within-class mediating weight, the conditional slope was $a_{Aj}^{W}=0.41+0.13\;Mas_{j}-0.09\;Per_{j}+u_{aj}$, with γ_*a*1_ = 0.13 (*p* < 0.001) and γ_*a*2_ = −0.09 (*p* = 0.004). A mastery climate one standard deviation above the grand mean amplified the within-level autonomy path by roughly 32%, whereas a one-standard-deviation increment in performance climate attenuated it by about 22%, both directions consistent with Hypotheses 2 and 3 (Jiang and Zhang, 2021). For the second-stage path from autonomy need satisfaction to intrinsic motivation, only mastery climate carried a significant moderating coefficient, γ_*b*1_ = 0.07 (*p* = 0.018), while the performance-climate term did not reach conventional significance, γ_*b*2_ = −0.03 (*p* = 0.210).

Substituting the conditional first-stage slope into the indirect-effect expression and evaluating the result at the four corner combinations of one standard deviation on each climate variable produced the following conditional indirect effects through autonomy, each with a Monte Carlo interval from 20,000 draws: 0.232, 95% CI [0.181, 0.291], under high mastery and low performance; 0.169, 95% CI [0.124, 0.220], under high mastery and high performance; 0.124, 95% CI [0.092, 0.162], under low mastery and low performance; and 0.069, 95% CI [0.044, 0.099], under low mastery and high performance. The index of moderated mediation for mastery climate, defined as the partial derivative of the conditional indirect effect with respect to the moderator at the mean of the other, was 0.061 with a 95% interval of [0.034, 0.094]; the corresponding index for performance climate was −0.038, 95% CI [−0.067, −0.012]. Both intervals exclude zero, and the joint operation of the two moderators produced a 3.4-fold spread between the most and the least favorable climate corners.

Figure 5 illustrates the climate-specific mediated component of predicted intrinsic motivation. The steepest slope occurs under high mastery and low performance, and the shallowest under low mastery and high performance. A follow-up specification testing for curvilinearity added a quadratic mastery-climate term to the slopes-as-outcomes equation. The quadratic coefficient was small but significant, γ_*a*1, 2_ = −0.04 (*p* = 0.041), indicating that the amplifying effect of mastery climate tapers as climate scores approach the upper third of the observed distribution. Taken together, the moderation results describe a graded rather than a threshold boundary condition, with the largest conditional indirect effect in classes that combine a high mastery emphasis with low performance pressure and the smallest where those signals are inverted.

**Figure 5:**
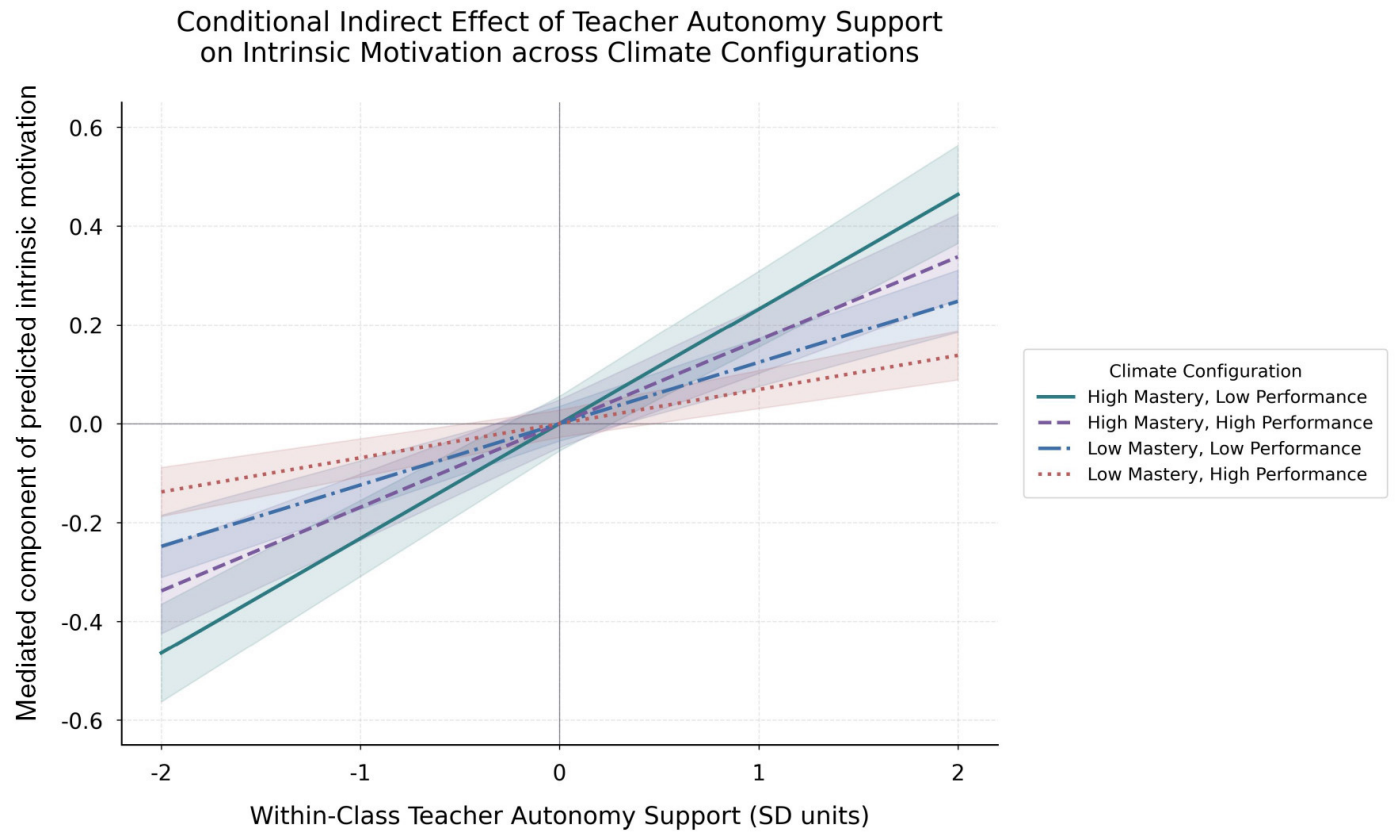
Simple-slope plots of the conditional within-class indirect effect from teacher autonomy support to intrinsic motivation, evaluated at the four combinations of mastery and performance climate at one standard deviation above and below the grand mean. Shaded bands depict 95% Monte Carlo confidence regions. Climate-specific mediated component of predicted intrinsic motivation through autonomy need satisfaction, relative to mean within-class autonomy support. Curves are shown for mastery and performance climates one standard deviation above or below their grand means. Their slopes represent the corresponding conditional indirect-effect estimates. Shaded bands represent 95% Monte Carlo intervals.

## 5 Discussion

The first research question asked whether the tripartite need-satisfaction mediation survives once between-class variance is properly partitioned, and the answer is that it does, at both levels and with paths that are neither redundant nor proportional. At the individual level the indirect effects through the three needs were modest but significantly non-zero, capturing how a student's idiosyncratic reading of her teacher, relative to her classmates' shared reading, translates into felt motivation. At the class level the same paths roughly doubled in magnitude, and the proportion of explained variance in motivation rose from 0.31 to 0.47. The within-level path plausibly reflects person-specific perceptual filters and dyadic teacher-student attunement, whereas the between-level path absorbs something the literature has been slower to name: the diffuse atmospheric quality of a class, its co-experienced norms, and the residue of repeated feedback episodes that no individual student fully owns. Single-level analyses, by collapsing the two, are liable to underestimate the contextual dividend that accrues when autonomy-supportive practice is consistent enough to register as a class-wide feature.

That reading of the level difference should not be adopted without competitors, and several deserve explicit consideration. Common method variance is the most obvious: all constructs came from the same student questionnaire on a single occasion, and although the Harman test and the latent-method-factor analysis reported in Section 4.1 found little evidence of mono-method inflation overall, neither procedure is well suited to detecting bias that operates at the class level. Shared response tendencies are a second candidate. Students in the same room are exposed to one another's evaluations of the teacher and may converge on a common frame of reference, which would inflate the between-class covariance among all self-reported constructs without any corresponding difference in experience. A third possibility is compositional: classroom characteristics that we did not model, among them teacher experience, class size, tracking status, and the prior achievement composition of the room, could drive both aggregated autonomy support and aggregated motivation. Because the design is cross-sectional and observational, the contextual effect reported here is best read as an upper bound on the class-level mechanism rather than as an estimate of it.

The second research question concerned the relative weight of the three needs, and here the formal contrasts require a more cautious statement than the point estimates alone would invite. Within classes, autonomy carried the heaviest mediating load, but it separated significantly only from relatedness; its margin over competence was marginal, and competence and relatedness were statistically indistinguishable. Between classes the differences narrowed further, and the joint test of equality was only borderline. Two readings fit the data. The first treats the cross-level shift as evidence that autonomy is the proximal nutrient at the dyadic margin while relatedness is partly a class-shared resource depending on collective teacher-student rapport. The second, more culturally inflected, notes that in densely scheduled Chinese secondary classrooms, where class identity is intense and peer attachment high, relatedness may be amplified at the contextual level by structural features that exist independently of any single teacher behavior. Either way, the findings argue against collapsing the three needs into a composite, since aggregation would have masked precisely the compensatory dynamics that make competence and relatedness worth modeling separately.

One qualification belongs with that conclusion. Because the outcome sits at the self-determined end of the regulation continuum, the autonomy path enjoys a conceptual proximity to intrinsic motivation that the other two mediators do not, a proximity that becomes explicit when autonomous functioning is indexed by a relative autonomy composite containing interest items (Ryan and Connell, 1989). We kept the constructs operationally separate and reported discriminant-validity evidence for the critical pair, yet the theoretical adjacency remains, and it is one reason to treat the autonomy advantage reported here as suggestive rather than settled. Because Section 2.1 frames the autonomy need as the mechanism rather than an early appearance of the outcome, this qualification remains a matter of interpretive caution rather than of construct redundancy: the mediator is the felt volition that accompanies acting, and the outcome is the self-determined quality of the reason for acting.

These results also bear on the universality thesis, though less decisively than the raw ordering might suggest. The qualitative ranking within classes, with autonomy first, matches the pattern commonly reported in Western samples, and the cultural inflection appears at the contextual level, where relatedness assumes greater weight than the canonical theory anticipates. That reading is consistent with the “universal-with-cultural-specificity” position on SDT in East Asian education (Yu et al., 2018a) and with meta-analytic evidence that the autonomy-well-being association is of comparable size across the two cultural regions (Yu et al., 2018b; Vite et al., 2024). We would nevertheless resist a strong claim on this point, because the contrasts in Table 6 show that the between-level ordering is not firmly established statistically; the correspondence to previously reported patterns is an observation about point estimates, not a demonstration that the needs differ in that order at the class level.

The third research question asked whether the classroom climate bends the mediated paths, and the asymmetric moderation we obtained is, in practical terms, the most consequential of the three findings. Mastery climate amplified the first stage of the chain, from teacher behavior to felt autonomy, and performance climate attenuated it; neither moderator did much to bend the second stage from need satisfaction to motivation. Extending the analysis to all three mediators clarified the boundary of that effect: the same pattern held for competence in weaker form, while relatedness proved insensitive to climate altogether, which suggests that the evaluative texture of a room shapes how agentic and competence-relevant cues are decoded more than it shapes the experience of belonging. The conditional indirect effect spanned a more than threefold range across climate corners, and the small but significant quadratic term points to diminishing returns once mastery emphasis exceeds typical levels. The message is uncomfortable for a one-size-fits-all reading of autonomy support: identical teacher behaviors yield substantially different motivational dividends depending on the evaluative surround.

The developmental gradient in perceived autonomy support deserves separate comment, both because it was statistically significant and because it bears on the decision to model six grades together. Seventh-graders reported the most autonomy-supportive teaching and twelfth-graders the least, with a partial rebound at the senior-secondary transition. Scalar invariance across grade bands and the consistency of the structural estimates when grade was entered as a covariate indicate that the mediation operates in much the same way across the span, so pooling the cohorts does not obscure heterogeneity in the process; what varies is the level of the predictor rather than the shape of the mechanism. Why the level should decline is a further question. Younger adolescents typically remain with a single homeroom teacher for more of the school day, which affords the sustained contact from which perspective-taking and affect acknowledgment become visible, whereas senior classes are organized around subject specialists and an examination calendar that compresses the room for choice, a pattern consistent with evidence that school level conditions the effect of perceived autonomy support (Xu et al., 2025) and that adolescents' readings of the school environment shift as they move upward (Wang and Holcombe, 2010). Declining perceived support may therefore index a structural change in teacher-student contact as much as a change in teacher intent, and a design that measured teacher behavior independently of student report would be needed to separate the two.

Three practical implications follow. Teacher training should target the specific behavioral facets of autonomy support, choice provision and rationale giving in particular, rather than promoting an abstract autonomy-supportive disposition, since it is the facets and not the disposition that students observe, and the teacher characteristics that sustain those practices are themselves trainable rather than fixed (Bardach et al., 2022). School leaders, for their part, should treat the building of classroom culture as a complement to individual teacher development, because without a mastery-leaning evaluative texture even capable teachers will see their efforts attenuated. And education policy that intensifies performance comparison risks closing the very motivational pipeline that autonomy-supportive practice opens, a tension worth acknowledging rather than papering over.

## 6 Conclusion

This study set out to map how teacher autonomy support shapes adolescent intrinsic motivation through basic psychological need satisfaction, treating the classroom not as a backdrop but as a structuring level of analysis. Drawing on responses from *N* = 2,634 students nested in *N* = 72 secondary classes, a two-level structural equation model with random slopes and slopes-as-outcomes moderation produced three interlocking conclusions. The mediated chain held at each level, but not to the same degree: the class-level paths ran about double their individual-level counterparts, and explained variance in motivation was 31% within classes and 47% between classes. Among the three needs, autonomy carried the heaviest mediating weight, although formal contrasts established its advantage over relatedness more firmly than over competence and left competence and relatedness statistically indistinguishable at both levels. Mastery-oriented climate amplified the first stage of the mediation by approximately a third and performance-oriented climate attenuated it by roughly a fifth, with a 3.4-fold spread between the most and least favorable climate corners; the same moderation appeared in weaker form for competence and not at all for relatedness.

Two contributions follow. Methodologically, the study moves the SDT classroom literature from single-level mediation toward properly partitioned multilevel mediation with explicit cross-level moderation and formal comparison of competing mediators, which sharpens what can and cannot be inferred from individual self-reports of teacher behavior. Theoretically, treating motivational climate as a first-stage moderator rather than an additive predictor refines the contextual specification of SDT and integrates it more cleanly with achievement goal theory. The practical implication, developed in Section 5, is that the returns to teacher training depend on the evaluative texture of the classes in which trained teachers work.

Several limitations qualify these conclusions. The cross-sectional design precludes strong causal inference about the temporal ordering of autonomy support, need satisfaction, and motivation. All constructs relied on student self-report, leaving room for shared-method variance at the class level even after the diagnostic checks reported in Section 4.1. We measured need satisfaction but not need frustration, so the asymmetric dual-process predictions noted in Section 2.1 remain untested here. The conceptual adjacency between autonomy satisfaction and intrinsic motivation, discussed in Section 5, means that the observed ordering of mediators should be replicated with outcomes further removed from the autonomy construct before it is treated as established. Measurement invariance was established across grade bands rather than across individual grades, and cross-cultural invariance was not examined at all. Finally, the sample, although large, was drawn from two prefecture-level cities in north-eastern China and may under-represent rural and minority-concentrated regions where the cultural inflection of relatedness might differ further. Future work could pursue four extensions: longitudinal panel designs to test temporal precedence; daily-diary methods to capture the within-person dynamics of autonomy experiences across lessons; observational or physiological indicators to triangulate the felt-volition construct beyond self-report; and teacher-student paired designs combined with cross-cultural sampling to disentangle the universal from the culturally specific layers of the climate-conditioned mediation observed here.

## Data Availability

The original contributions presented in the study are included in the article. Further inquiries can be directed to the corresponding author.
